# Linking Temperature, Cation Concentration and Water Activity for the B to Z Conformational Transition in DNA

**DOI:** 10.3390/molecules23071806

**Published:** 2018-07-21

**Authors:** Jaime M. Ferreira, Richard D. Sheardy

**Affiliations:** 1 Estee Lauder Companies, Inc., Melville, NY 11747, USA; jferrier@estee.com; 2 Department of Chemistry and Biochemistry, Texas Woman’s University, Denton, TX 76204, USA

**Keywords:** B-DNA, Z-DNA, circular dichroism, calorimetry, enthalpy, conformational transitions, heat capacity

## Abstract

High concentrations of Na^+^ or [Co(NH_3_)_6_]^3+^ can induce the B to Z conformational transition in alternating (dC-dG) oligo and polynucleotides. The use of short DNA oligomers (dC-dG)_4_ and (dm^5^C-dG)_4_ as models can allow a thermodynamic characterization of the transition. Both form right handed double helical structures (B-DNA) in standard phosphate buffer with 115 mM Na^+^ at 25 °C. However, at 2.0 M Na^+^ or 200 μM [Co(NH_3_)_6_]^3+^, (dm^5^C-dG)_4_ assumes a left handed double helical structure (Z-DNA) while the unmethylated (dC-dG)_4_ analogue remains right handed under those conditions. We have previously demonstrated that the enthalpy of the transition at 25 °C for either inducer can be determined using isothermal titration calorimetry (ITC). Here, ITC is used to investigate the linkages between temperature, water activity and DNA conformation. We found that the determined enthalpy for each titration varied linearly with temperature allowing determination of the heat capacity change (ΔC*_p_*) between the initial and final states. As expected, the ΔC*_p_* values were dependent upon the cation (i.e., Na^+^ vs. [Co(NH_3_)_6_]^3+^) as well as the sequence of the DNA oligomer (i.e., methylated vs. unmethylated). Osmotic stress experiments were carried out to determine the gain or loss of water by the oligomer induced by the titration. The results are discussed in terms of solvent accessible surface areas, electrostatic interactions and the role of water.

## 1. Introduction

The determination of the structure of B-DNA as a right-handed double helical structure in 1953 by Watson and Crick [1] was just the beginning of structural studies on DNA. Deviations from pure B-DNA, such as A-DNA, C-DNA, and Z-DNA, have revealed that DNA is highly polymorphic and that the exact secondary structure of a segment of DNA is highly dependent upon both the sequence context and the local environment. Although most DNA are considered to adopt a right-handed double helical conformation, the observation of an inverted circular dichroism spectrum of poly(dC-dG) in high salt led Pohl and Jovin to propose a left-handed double helical conformation for that polymer under those conditions [2]. The ability of DNA to adopt a left-handed double helical conformation was confirmed by X-ray crystallography in 1979 when Rich et al. determined the structure of an alternating (GC) oligomer in the presence of Mg^2+^ and Co^3+^ to be a left-handed [3]. This conformation was designated as Z-DNA due to the “zig-zag” arrangement of the sugar-phosphate backbone. The ability of DNA to undergo the B to Z transition is not only influenced by sequence and environmental effects but also by modification of the DNA itself. For example, Behe and Felsenfeld compared poly(dC-dG) to poly(dm^5^C-dG) for their abilities to undergo B to Z transitions using Na^+^, Mg^2+^ or trivalent cobalt hexamine [Co(NH_3_)_6_]^3+^ and found that: (1) the transition occurred at a much lower concentrations of inducer for the methylated polymer; and, (2) the concentration of the inducer used at the midpoint of the transition decreased with increasing charge of the inducer [4].

The ability of Z-DNA to be formed under physiological salt conditions due to the methylation at the 5′ position has led to the study of the different roles Z-DNA may play biologically. DNA sequences that are either in a Z conformation or go through a transformation to become Z DNA have been identified in vitro, in vivo, and in eukaryotic systems [5,6,7]. It has been shown that Z-DNA binding proteins known as anti-Z-DNA antibodies induce Z-DNA formation and help stabilize left-handed DNA in vivo [8]. It has also been suggested that Z-DNA binding proteins are actually phospholipid-binding proteins, or that Z-DNA binding proteins regulate gene expression by turning genes on and off [9]. Z-DNA was first observed in chromosomes of the fruit fly *Drosophila melanogaster* by Rich et al. and was shown to be present in fixed and unfixed tissue sections by immunohistochemical methods [10]. These studies revealed that the B to Z transition in ds-DNA molecules is caused by the torsional stress build up related to the Z-DNA’s immunoreactivity.

Regardless of the biological role of Z-DNA, sequences that undergo the B to Z transition offer robust models to study the thermodynamics of conformational transitions. Using van’t Hoff analysis, Pohl and Jovin determined the enthalpy of the Na^+^ induced B to Z transition of poly(dC-dG) to be near zero (±1 kcal/mol bp) and temperature independent over the range of 30 to 50 °C [2]. Using differential scanning calorimetry, Chaires and Sturtevant reported an enthalpy of 0.61 ± 0.07 kcal/mol bp for the B to Z transition of poly(m^5^dC-dG) [11]. For the unmethylated polymer, Chaires and Sturtevant reported an enthalpy of 2.02 ± 0.2 kcal/mol bp [12] in excellent agreement with the enthalpy reported independently by Klump, et al. [13]. We have investigated the B to Z transition of (m^5^dC-dG)_4_ induced by either Na^+^ or [Co(NH_3_)_6_]^3+^ at 25 °C using isothermal titration calorimetry. We found the enthalpy of the B to Z transition to be 0.70 ± 0.04 kcal/mol bp for either inducer [14]. A molecular dynamics approach also suggested that the B to Z transition resulted in a ΔH of 0.9 kcal/mol, in good agreement with previous experimentally derived values [15].

Manzini et al. suggested that in high salt the B to Z conversion proceeds through denaturation of the duplex with B to coil to Z [16], while Tran-Dinh and others proposed a Z-to-B to coil mechanism in which a direct Z-DNA helix to single-strand transition was not observed [17]. Results from our group suggests that the B to Z transition proceeds through three states: B to I to Z, where I is most likely an intermediate dehydrated structure [14,18].

For our studies, we used two self-complementary eight base pair DNA oligomers to monitor the B to Z transition: Z8A, (dC-dG)_4_, and Z8M, (dm^5^C-dG)_4_. To expand upon our previous study, we have investigated the influence of temperature and water activity on the B to Z transition using calorimetric approaches. Isothermal titration calorimetry (ITC) has proven to be a reliable tool to determine enthalpy values of conformational changes in macromolecules. ITC measurements allow a direct and sensitive determination of the transition enthalpy, and provide a significant advantage over methods based on the indirect van’t Hoff analysis.

## 2. Results and Discussion

### 2.1. The Enthalpy of the B to Z Transition

We have previously shown that the addition of either Na^+^ or [Co(NH_3_)_6_)]^3+^ induces the B to Z transition for Z8M but not for Z8A based on circular dichroism (CD) spectra. Under low salt concentrations (i.e., 115 mM Na^+^), the CD spectra of both Z8M and Z8A are consistent with right-handed B conformations with peaks at 280 nm and troughs at 255 nm. Under high salt conditions (i.e., 2.0 M Na^+^ or 200 mM [Co(NH_3_)_6_]^3+^), Z8A remains right handed (the CD spectra under low salt and high salt conditions are superimposable) but Z8M assumes a left handed Z conformation with a peak at 278 nm and a trough at 295 nm (the CD spectra under high salt conditions are “inverted” from those under low salt conditions) [14]. Similar results were obtained for all studies presented here (data not shown).

The B to Z transition enthalpy has previously been obtained by a variety of experimental techniques [2,11,12,13,19,20,21]. As demonstrated in our earlier report [14] and here, the transition enthalpy can also be determined using Isothermal Titration Calorimetry (ITC) by assuming that the difference in total enthalpy between Z8A and Z8M upon addition of either Na^+^ or [Co(NH_3_)_6_]^3+^ is due to the transition itself. In other words, for any oligomer undergoing a salt titration:

ΔH_*obs*_ = ΔH_*conf*_ + ΔH_*nonconf*_ (1)

where ΔH*_obs_* is the observed calorimetric enthalpy, ΔH*_conf_* is the enthapy for the B to Z conformational transition itself and ΔH*_nonconf_* is the enthalpy for all other possible processes such as uptake or loss of Na^+^, uptake or loss of water, and binding of [Co(NH_3_)_6_]^3+^ when titrating with this inducer. The governing premises here are: (1) that ΔH*_nonconf_* is the same for Z8A and Z8M with either Na^+^ or [Co(NH_3_)_6_]^3+^; and, (2) that ΔH*_conf_* is essentially zero for Z8A since it does not undergo the B to Z transition with either inducer [14]. This leads to:

ΔH_*conf*_ = ΔH_*obs,Z8M*_ − ΔH_*obs,Z8A*_ (2)

Thus, both Z8A and Z8M were titrated with either Na^+^ or [Co(NH_3_)_6_]^3+^ and the enthalpies of the titrations determined. 

**Figure 1:**
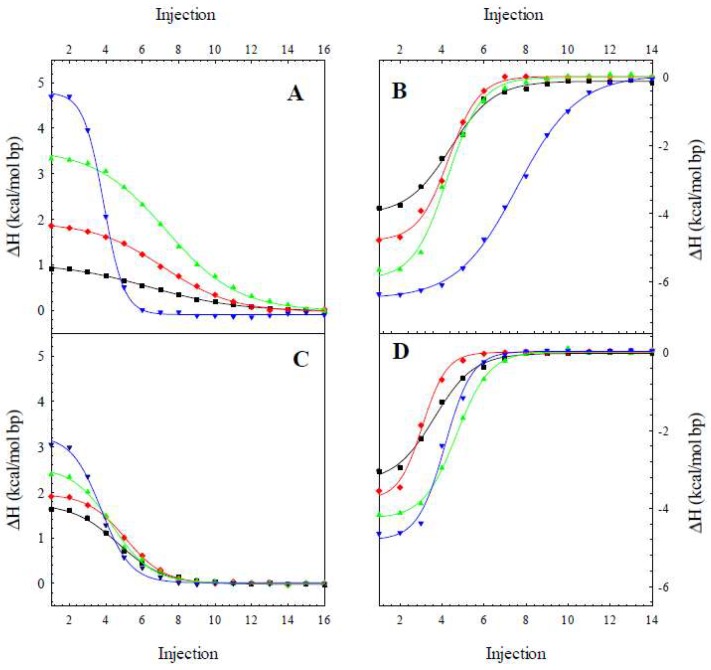
ITC Binding isotherms obtained for (**A**) Z8A and NaCl; (**B**) Z8A and [Co(NH_3_)_6_]^3+^; (**C**) Z8M and NaCl; and (**D**) Z8M and [Co(NH_3_)_6_]^3+^ interactions as a function of temperature: 25 °C (black), 35 °C (red), 45 °C (green), and 55 °C (blue). The integrated results of the data after subtraction of the heats of dilution are represented and the solid lines are the best least-square fits of the integrated data.

It was first observed that the heat of dilution Na^+^ is endothermic while that of [Co(NH_3_)_6_]^3+^ is exothermic at 25 °C [14]. This difference is most likely due to differences in hydration of these ions. Examination of typical raw calorimetric titration data at 25 °C reveals that titration of either Z8M or Z8A with Na^+^ is also endothermic while titrations with [Co(NH_3_)_6_]^3+^ are exothermic [14]. The titrations were then carried out at temperatures up to 55 °C. Subtraction of the heats of dilution from the respective DNA titrations, followed by integration of the data results in the isotherms shown in Figure 1. Not only are the titrations of Z8A or Z8M with Na^+^ endothermic but also become more endothermic as the temperature increases from 25 to 55 °C. Further, the titrations of these oligomers with [Co(NH_3_)_6_]^3+^ become more exothermic with increasing temperature. These trends are strictly consistent with Le Châtelier’s principle. One final observation from Figure 1 is that the transitions appear to become more cooperative at higher temperatures. The ITC determined enthalpy values obtained from these titrations can be found in Table 1. What is particularly noteworthy, however, about that data in Table 1 is that the B to Z transition enthalpy (ΔH*_conf_* values) induced by Na^+^ becomes more favorable with increasing temperature while the [Co(NH_3_)_6_]^3+^ induced transition becomes less favorable with increasing temperature. This observation will be addressed in the next section.

**Scheme 1:**
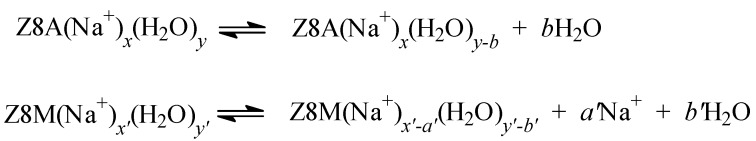
The net reaction for the titration of Z8A or Z8M with Na^+^. Upon addition of Na^+^ to Z8A, *b* moles of water are released into the bulk. In the titration of Z8M with Na^+^, the B to Z conformational change due to the dehydration of the oligomer gives rise to chain lengthening. The chain lengthening lowers the overall charge density of the oligomer thereby allowing release of Na^+^ into the bulk as well.

**Scheme 2:**
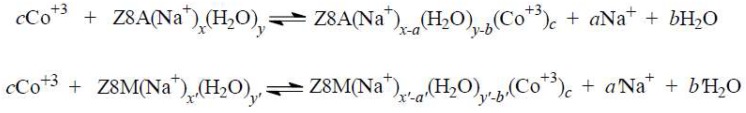
The net reaction for the titration of Z8A or Z8M with [Co(NH_3_)_6_]^3+^. In the titrations of either Z8A or Z8M with [Co(NH_3_)_6_]^3+^ both water, due to the dehydration, and Na^+^, due to the binding of the cobalt complex, are released from the oligomers into the bulk solution. However, the number of moles of water (*b* and *b*’) and moles of Na^+^ (*a* and *a*’) released, respectively, are different for the different oligomers due to the conformational change of Z8M (i.e., *b* ≠ *b*’ and *a* ≠ *a*’).

Our transition enthalpy of 0.71 kcal/mol bp obtained at 25 °C using Na^+^ compares quite favorably with the 0.61 kcal/mol bp reported by Chaires and Sturtevant [11], although obtained by different techniques (i.e., ITC vs. DSC). To rationalize the relative signs and magnitudes of the enthalpy values at 25 °C for the systems studied here, one can consider the equilibria shown in Scheme 1 and Scheme 2. In the titration of either Z8A or Z8M with Na^+^, the net reaction can be described by the conversion of Z8A from a true B-conformation to a B’-conformation (the dehydrated form indicated as I above) or the conversion of Z8M from a true B-conformation to a Z-conformation, both with concomitant loss of H_2_O. The increase in ionic strength upon addition of Na^+^ to either DNA leads to its dehydration. Z8M is initially less hydrated than Z8A due to the presence of the methyl groups in the major groove. At the end point of the titration, i.e., 2.0 M Na^+^, Z8M is thus even more dehydrated the Z8A. In the case of Z8M, its dehydration leads to the B to Z conformational transition. The loss of water from either oligomer is clearly enthalpically unfavorable. Further, Z-DNA has a lower charge density than B-DNA and thus requires fewer associated Na^+^. Hence, Z8M also experiences a net loss in Na^+^, also enthalpically unfavorable, as it undergoes the transition. Thus, increasing the [Na^+^] from 115 mM to 2.0 M should be enthalpically unfavorable and more so for Z8M than for Z8A, as observed.

In the titrations of Z8A and Z8M with [Co(NH_3_)_6_]^3+^, one must also take into account the binding of the cobalt complex to the DNA (Scheme 2). This enthalpically favorable binding is due, in part, to the formation of five hydrogen bonds from the cobalt complex to the surface of the DNA [22]. This binding also leads to the loss of bound Na^+^ [23,24] and H_2_O from both oligomers. Apparently, the unfavorable loss of Na^+^ and H_2_O is compensated by the favorable binding of the cobalt complex. In this scenario, the titration of Z8M is less favorable than that for Z8A due to the conformational transition of Z8M.

### 2.2. The Heat Capacity Change for the B to Z Transition

**Figure 2:**
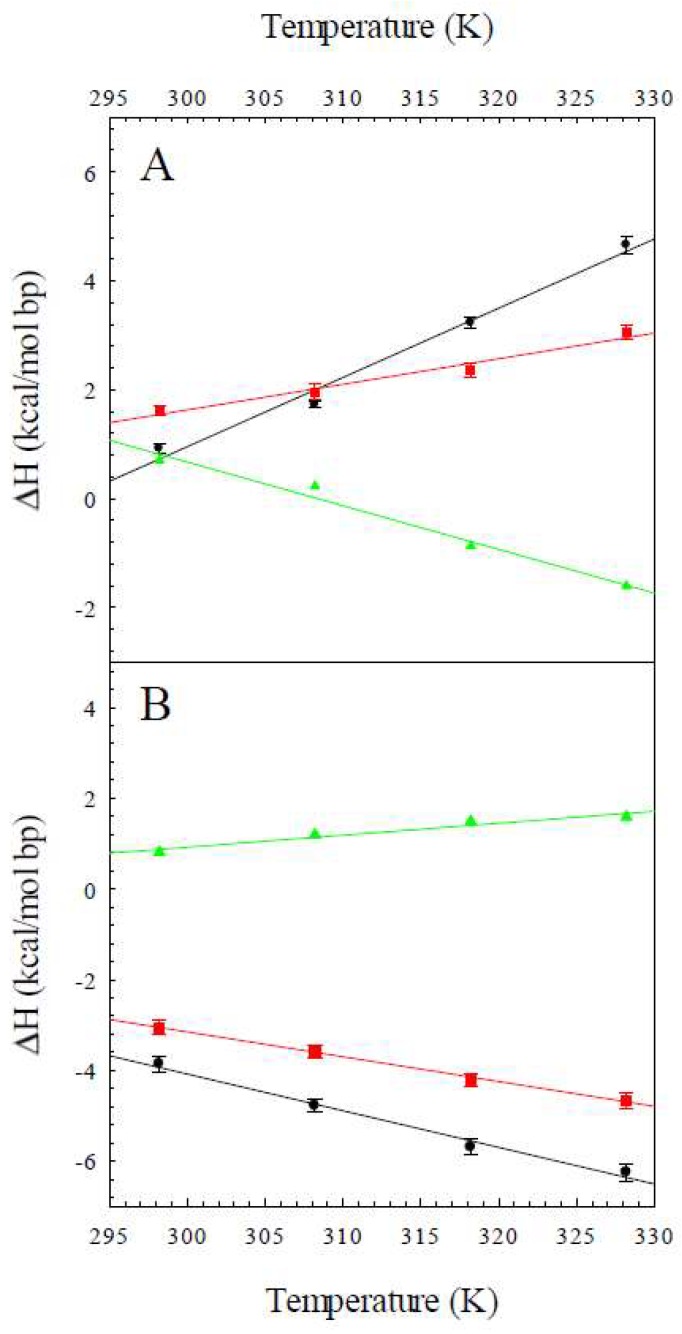
Plots of ΔH*_obs_*_,*Z8A*_ (black), ΔH*_obs_*_,*Z8B*_ (red) and ΔH*_conf_* (green), calculated using Equation (2), versus temperature for the titrations with (**A**) Na^+^ or (**B**) [Co(NH_3_)_6_]^3+^. The solid lines are the least squares linear fits and the slopes of those lines yield the difference in heat capacities, ΔC*_p_*, of the DNA oligomers between the initial state to the final state. These values can be found in Table 2.

As can be seen in Figure 2, the enthalpies of all titrations vary linearly with increasing temperature albeit with different slopes. As a result of these nonparallel slopes for the titrations of Z8A and Z8M, the magnitude and, in the case when titrating with Na^+^, the sign of the enthalpies of the B to Z transitions (ΔH*_conf_*) are also temperature dependent as noted above and in Table 1.

The slope of the resultant least squares linear fit corresponds to ΔC*_p_*, the difference in the heat capacity of the oligomer under different environmental conditions. These ΔC*_p_* values are listed in Table 2. Increasing the concentration of Na^+^ from 115 mM to 2.0 M results in an increase in the heat capacities of both Z8A and Z8M by 127 and 47 cal/K mol bp, respectively. On the other hand, increasing the concentration of [Co(NH_3_)_6_]^3+^ from 0 to 200 μM decreases the heat capacities of both Z8A and Z8M by 55 and 81 cal/K mol bp, respectively. As a point of reference, ΔC*_p_* values for DNA denaturation have been reported to range from 40 to 100 cal/K mol bp for DNA polymers [25,26], up to1.3 kcal/K mol duplex for DNA oligomers [27] and 20 to 30 cal/K mol bp for a DNA oligomer which forms the *i*-motif [28]. Hence the heat capacity changes observed here for conformational transitions in short DNA duplexes are similar in magnitude to those observed for other conformational transitions.

It has been shown that changes in heat capacities for biomolecules undergoing conformational changes or ligand binding can partly be attributed to changes in solvent accessible surface areas [29,30,31,32,33,34,35,36,37,38,39]. Certainly, the Z-DNA conformation has a quite different solvent accessible surface area than the B-conformation. The dehydrated B-conformation should also have a different solvent accessible surface than the normal hydrated B-form. Using parameters derived from crystal structures of d(CpG)_3_ and d(5mCpG)_3_, calculations of the average solvent accessible surface areas (SAS) per base pair reveal only a slight difference in the SAS for the B and Z forms of unmethylated DNA (272.8 Å^2^ vs. 275.4 Å^2^, respectively) but a much larger difference in the SAS for the B and Z forms of methylated DNA (295.5 Å^2^ vs. 263.1 Å^2^, respectively) [40]. Further, the hydrations energies are much lower for the B and Z conformations of the unmethylated base pair than for the methylated base pair [40].

The presence of the cobalt complex bound to the DNA surface should also occlude solvent molecules. The positive heat capacity changes observed in the titrations of either DNA oligonucleotide with Na^+^ can be attributed to burial of hydrophobic surfaces and/or exposure of hydrophilic surfaces [27,30,31,32,33]. This is quite reasonable considering the 10 fold increase in ionic strength throughout the titration. The negative heat capacity changes observed in the titrations with [Co(NH_3_)_6_]^3+^ can be partially attributed to the decrease in exposed hydrophilic surface due to the bound cobalt complex. It should be noted, however, that heat capacity changes may also be due to factors other than changes in solvent accessible surface area, particularly for nucleic acids [31,32,35,36,38,39]. It has been suggested that the observed sequence and salt dependent ΔC*_p_* associated with duplex formation reflect perturbations to base stacking in the single strand [36]. Base stacking in the duplex accompanying a conformational transition may also contribute to changes in heat capacity. Ultimately, the changes in heat capacities observed here are due to a combination of changes in solvent accessible surface areas, conformational changes and electrostatic interactions. All of these factors will affect the number of water molecules released upon addition of Na^+^ or [Co(NH_3_)_6_]^3+^ and therefore, influence the relative values of *b* and *b*’ for both titration with Na^+^ and titration with [Co(NH_3_)_6_]^3+^ (Scheme 1 and Scheme 2).

### 2.3. The Release of Water

The role of water, or more specifically, water activity on the stability of DNA conformations and their ligand binding properties, has been the focus of many studies through the utilization of osmotic stress investigations [41]. Consideration of the Scheme 1 and Scheme 2 indicate that the release of water during the titrations must also be considered even in the absence of a transition. Clearly, increasing the concentration of Na^+^ or [Co(NH_3_)_6_]^3+^ will alter the activity of water. Preisler et al. [42] examined the effects of neutral solutes on the B to Z transition of poly (dG-dC). Their results suggested that the solute effects were not consistent with direct binding of the solute to the DNA nor was there an indirect effect on electrostatics or with alterations in ion binding due to changes in solution dielectric. In other words, both the solute and NaCl stabilize Z DNA through osmotic stress. Further, they estimated that the number of water molecules released during the B to Z transition to be ~2.5 per base pair as probed in sucrose. However, for any particular osmolyte, the release of water will depend upon its size and chemical nature.

**Figure 3:**
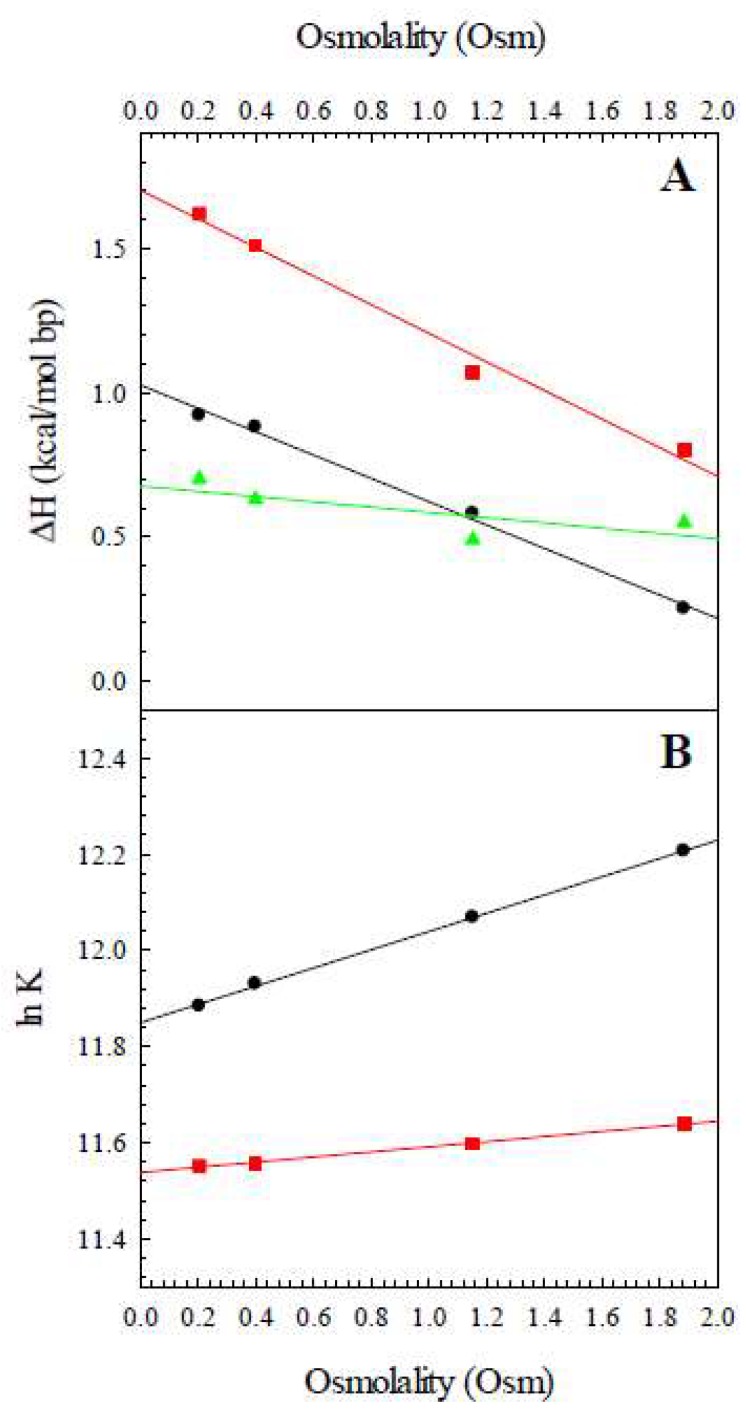
(**A**) Plots of ΔH*_obs_*_,*Z8A*_ (black), ΔH*_obs_*_,*Z8B*_ (red) and ΔH*_conf_* (green), calculated using Equation (2), versus osmolality for the titrations with Na^+^ using betaine as the osmolyte. (**B**) Plots of lnK vs. osmolality for Z8A (black) and Z8M (red).

The Na^+^ titrations of Z8M and Z8A in the presence of the neutral osmolyte, betaine, were examined by ITC. Plots of ΔH vs. osmolality of the DNA solution in the absence or presence of betaine resulted in linear correlations as depicted in Figure 3A. As can be seen, the osmolyte lowered the enthalpy for the titration for both Z8M and Z8A; however, the difference in enthalpies between Z8M and Z8A, ΔH*_conf_*, remains fairly constant at all osmolalities suggesting that the enthalpy of the B to Z transition is independent upon the concentration of betaine, as it should be [43,44]. 

Applying the linkage relationship developed by Wyman and others [43,44,45,46,47], one can write

∂∆G^o^/RT∂ln a*_w_* = ∆n*_w_* (3)

where a*_w_* is the activity of water and Δn*_w_* is the differential water binding term, i.e, the number of water molecules released or taken up by the oligomer during the transition. Using the relationship that ΔG^o^ = −RTlnK and ln a*_w_* = −Osm/55.55, where Osm is the osmolality of the solution, Equation (3) becomes:

∆n*_w_* = 55.55 ∂lnK/∂Osm (4)

As can be seen in Figure 3B, there is a linear relationship between ln K and osmolality for the titrations of Z8A and Z8M in the presence of the osmolyte betaine. The slopes of the resultant least squares regression allows determination of the coefficients *b* and *b*’ of Scheme 1 of 2.9 molecules bp^−1^ and 10.6 water molecules bp^−1^ for Z8A and Z8M respectively. Thus, the conformational B to Z transition leads to the release of an additional 7.7 molecules of water per base pair. Since changes in heat capacities have been related to loss or gain of water, it is interesting that that Z8M, which has the largest loss of water, also has the smallest change in heat capacity (Table 3).

Our value of 10.6 water molecules per base pair is much higher than the 2.5 water molecules per base pair reported by Preisler for poly(dG-m^5^dC) in the presence of sucrose as the osmolyte [42]. Both Z8M ((dG-m^5^dC)_4_) and the corresponding polymer (poly(dG-m^5^dC)) undergo the B to Z transition. The difference in released waters is likely due to the different sizes of the DNA (8-mer vs. polymer), different osmolytes (betaine vs. sucrose) and different Z inducer (Na^+^ vs. [Co(NH_3_)_6_]^3+^). The release or uptake of water by DNA has become a more important physical quantity as we learn more about the role of water in related chemical and physical processes. For a comparison, Δn*_w_* for the duplex or triplex to coil transition has been determined to be dependent upon DNA conformation, sequence context, and nature of added cosolute or osmolyte with values ranging from 0 to 27 water molecules per base pair, depending upon experimental conditions [46,47]. The folding of d(C_3_TA_2_)_4_ from the single strand to the *i*-motif upon addition of protons is accompanied by a release of only 0.3 mole H_2_O per/mol strands [48]. The uptake of water by the denaturation of DNA hairpins has been shown to range from 51 to 73 molecules of water per base pair [49]. For a final comparison, Son et al. [50] demonstrated that the denaturation of the duplex formed by the decamer (GGCATTACGG/CCGTAATGCC) is accompanied by the uptake of around 180 molecules of water per duplex, or about 18 molecules per base pair.

## 3. Experimental Section

### 3.1. Oligomer Design, Synthesis and Preparation

DNA oligonucleotides were synthesized and purified as previously described [14,51,52,53,54,55]. The DNA concentration and yield were determined by spectrophotometric absorbance using extinction coefficients, ε (L mol^−1^ cm^−1^ in base pairs), of 13,000 for Z8A and 13,620 for Z8M at 255 nm. The [Co(NH)_3_)_6_]Cl_3_ was obtained from Kodak (Rochester, NY, USA) and used without further purification. Lyophilized DNA samples were reconstituted in a 10 mM phosphate buffer (pH 7.0), 0.1 mM EDTA with NaCl or [Co(NH)_3_)_6_]Cl_3_ complex added to vary their concentrations. Samples were then heated to 90 °C for 2 min followed by slow cooling and equilibration for 48 h at 4 °C.

### 3.2. Isothermal Titration Calorimetry

Isothermal Titration Calorimetry measurements were carried out using the isothermal titration module of CSC Model 4200 ITC (Calorimetry Sciences Corp., Lindon, UT, USA). CSC Run, Bindwork™ and Origin 4.0 software were used for data acquisition and analysis as previously described [14]. Each experiment was set up such that 10 μL of 800 μM [Co(NH)_3_)_6_]Cl_3_ or 10 μL of 4 M NaCl was titrated into the sample cell containing either Z8A or Z8M at 115 μM duplex for up to a total of 25 injections.

The final concentrations of Na^+^ or [Co(NH)_3_)_6_]^3+^ were 2.0 M and 200 μM, respectively. Titrations were carried out at 25, 35, 45, and 55 °C, temperatures well below the T_m_ of the respective oligomers [14]. Control experiments were carried out to determine the contributions to the enthalpy from the heat of dilution for both the Na^+^ and [Co(NH)_3_)_6_]^3+^ into buffer or water, respectively. The net enthalpy for each injection was determined by subtraction of the component heats of dilution. The primary source of error in these determinations lies in the inherent uncertainty of the extinction coefficients used for the DNA oligomers. These values can vary by 5 to 10% depending on the method used for their determination. We used extinction coefficients which determined by a nearest neighbor approach.

### 3.3. Osmotic Stress Experiments

To investigate the role of water activity on the B to Z transition, DNA solutions were prepared as above with the addition of the osmolyte betaine and titrated as described. The osmolality of the buffer in the absence and presence of betaine was determined using an Advanced Instruments 3220 Osmometer which determines osmolality via freezing point depression.

## 4. Summary

We have used ITC approaches to investigate the conformational transitions of two related oligomers. The data obtained allowed determination of thermodynamic parameter ΔH, ΔC*_p_* and Δn*_w_* for each oligomer. The ITC studies indicated that the B to Z transition becomes more enthalpically favorable at higher temperatures using Na^+^ as an inducer but less enthalpically favorable using [Co(NH_3_)_6_]^3+^ as the inducer. These trends are due to differences in ΔC*_p_* values for each oligomer for a particular inducer. Further, the B to Z conformational transition leads to a larger loss of water from the oligomer. Ultimately, the observed thermodynamic parameters can be rationalized in terms of changes in solvent accessible surface area, uptake or release of Na^+^, release of water and binding of [Co(NH_3_)_6_]^3+^. Overall, this work demonstrates the utility of using ITC to investigate enthalpy changes due to conformational transitions, which may not be observable using only spectroscopic approaches.

## Figures and Tables

**Table 1:** Summary of ITC determined enthalpies for the titrations of Z8A and Z8M.

T (K)	ΔH*_obs_*_,*Z8A*_ (kcal/mol bp)	ΔH*_obs_*_,*Z8M*_ (kcal/mol bp)	ΔH*_conf_* (kcal/mol bp)
Na^+^			
298	0.92 ± 0.09	1.62 ± 0.09	0.70
308	1.73 ± 0.06	1.96 ± 0.14	0.23
318	3.23 ± 0.11	2.35 ± 0.12	−0.87
328	4.66 ± 0.15	3.06 ± 0.14	−1.6
[Co(NH_3_)_6_]^3+^			
298	−3.85 ± 0.17	−3.13 ± 0.15	0.72
308	−4.78 ± 0.13	−3.58 ± 0.15	1.2
318	−5.69 ± 0.18	−4.20 ± 0.15	1.5
328	−6.25 ± 0.18	−4.66 ± 0.19	1.6

Values were obtained from the total integrated isotherms shown in Figure 1. ΔH*_obs_*_,*Z8A*_ and ΔH*_obs_*_,*Z8M*_ are the observed enthalpies for the respective titrations of Z8A and Z8M and ΔH*_conf_* represents the enthalpy for the B to Z transition at the temperature indicated, i.e., Δ*H_conf_* = ΔH*_obs_*_,*Z8M*_ − ΔH*_obs_*_,*Z8A*_ as per Equation (2).

**Table 2:** The change in heat capacities (ΔC*_p_*) for of Z8A and Z8M resulting from their titrations with either Na^+^ or [Co(NH_3_)_6_]^3+^.

Inducer	Z8A (kcal/K mol bp)	Z8M (kcal/K mol bp)	Z8M–Z8A (kcal/K mol bp)
**Na^+^**	+0.127	+0.0469	−0.0802
**[Co(NH_3_)_6_]^3+^**	−0.0545	−0.0808	+0.0263

Values were obtained from the slopes of the least squares linear fits of the data in Figure 2.

**Table 3:** The change in enthalpies (ΔH) for of Z8A and Z8M resulting from their titrations with Na^+^ in the absence or presence of betaine.

Osmolality (Osm)	ΔH*_obs_*_,*Z8A*_ (kcal/K mol bp)	ΔH*_obs_*_,*Z8M*_ (kcal/K mol bp)	ΔH*_conf_* (kcal/K mol bp)
0.20	0.92	1.62	0.70
0.40	0.88	1.51	0.63
1.15	0.58	1.03	0.45
1.88	0.23	0.82	0.59

Values were obtained from the total integrated isotherms (data not shown). ΔH*_obs_*_,*Z8A*_ and ΔH*_obs_*_,*Z8M*_ are the observed enthalpies for the respective titrations of Z8A and Z8M and ΔH*_conf_* represents the enthalpy for the B to Z transition at the temperature indicated, i.e., Δ*H_conf_* =ΔH*_obs_*_,*Z8M*_ − ΔH*_obs_*_,*Z8A*_ as per Equation (2).
